# Exploration of Potential Broad-Spectrum Antiviral Targets in the Enterovirus Replication Element: Identification of Six Distinct 5′ Cloverleaves

**DOI:** 10.3390/v16071009

**Published:** 2024-06-23

**Authors:** Morgan G. Daniels, Meagan E. Werner, Rockwell T. Li, Steven M. Pascal

**Affiliations:** 1 Department of Chemistry and Biochemistry, Old Dominion University, Norfolk, VA 23529, USA; mdani006@odu.edu (M.G.D.); mwern002@odu.edu (M.E.W.); 2 Math and Science Academy, Ocean Lakes High School, Virginia Beach, VA 23454, USA; rockwell.t.li@gmail.com

**Keywords:** RNA, *enterovirus*, picornavirus, cloverleaf, replication

## Abstract

*Enterovirus* genomic replication initiates at a predicted RNA cloverleaf (5′CL) at the 5′ end of the RNA genome. The 5′CL contains one stem (SA) and three stem-loops (SLB, SLC, SLD). Here, we present an analysis of 5′CL conservation and divergence for 209 human health-related serotypes from the *enterovirus* genus, including enterovirus and rhinovirus species. Phylogenetic analysis indicates six distinct 5′CL serotypes that only partially correlate with the species definition. Additional findings include that 5′CL sequence conservation is higher between the EV species than between the RV species, the 5′CL of EVA and EVB are nearly identical, and RVC has the lowest 5′CL conservation. Regions of high conservation throughout all species include SA and the loop and nearby bases of SLB, which is consistent with known protein interactions at these sites. In addition to the known protein binding site for the Poly-C binding protein in the loop of SLB, other conserved consecutive cytosines in the stems of SLB and SLC provide additional potential interaction sites that have not yet been explored. Other sites of conservation, including the predicted bulge of SLD and other conserved stem, loop, and junction regions, are more difficult to explain and suggest additional interactions or structural requirements that are not yet fully understood. This more intricate understanding of sequence and structure conservation and variability in the 5′CL may assist in the development of broad-spectrum antivirals against a wide range of *enteroviruses*, while better defining the range of virus isotypes expected to be affected by a particular antiviral.

## 1. Introduction

**Figure 1:**
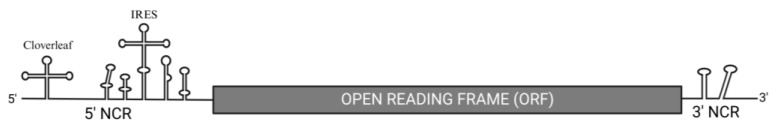
Block diagram of the picornavirus genome. The positive-sense single-stranded RNA genome contains a single open reading frame flanked by two non-coding regions (NCRs). The internal ribosome entry site (IRES) is needed to initiate translation of the open reading frame, while the 5′CL is critical for initiation of replication. Created with BioRender.com.

The *enteroviruses*, a genus in the picornavirus family, consist of ~300 virus types characterized by a small (~7.5 kb) positive-sense RNA genome [1,2]. Members of the *enterovirus* genus cause various human illnesses and conditions, including viral meningitis, polio, hepatitis, myocarditis, and the common cold [3,4,5]. The *enterovirus* RNA genome consists of three distinct regions (Figure 1): one open reading frame (ORF) flanked by two non-coding regions (NCRs). The ORF codes for a single viral polyprotein, approximately 2330 amino acids in length. The polyprotein is post-translationally cleaved by viral proteases to produce active virus proteins. A variety of partially-cleaved polyprotein states exist, some of which play essential roles in the replication process [6,7]. For example, the main viral protease (3C^pro^) can remain contiguous with the viral replicase (3D^pol^), forming the 3CD polyprotein, as is discussed further below.

The two NCRs, one 5′ and one 3′, flank the ORF and form key structural elements that play active roles in biological processes. These include an approximately 86-nucleotide cloverleaf structure at the extreme 5′-end of the genome (5′CL), followed by a type I internal ribosome entry site (IRES) containing approximately 450 nucleotides [8]. The 3′-NCR is much smaller, with 37–99 nucleotides, and includes a poly-A tail. While small, the 3′NCR contains essential hairpins (domain X and Y) that, through Watson–Crick base pairing, form a pseudoknot-like structure that is critical for the virus replication cycle [9]. In the 5′NCR, the IRES attracts host ribosomes, in the absence of the usual mRNA capping structure, triggering translation of the viral polyprotein. In fact, IRES motifs were first discovered in picornaviruses [8,10] and have since been found in the human genome [11].

**Figure 2:**
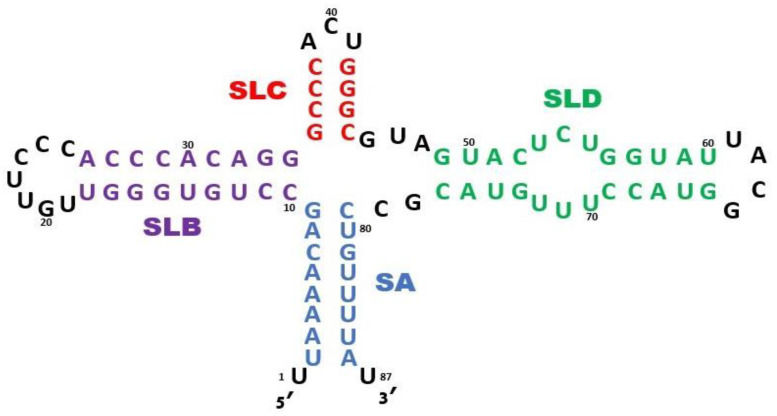
Secondary structure prediction for the *enterovirus* 5′CL consensus sequence. The predicted 5′CL contains a terminal stem (SA: blue) and three stem-loops (SLB: purple, SLC: red, and SLD: green). At times in the text, the four stems will be referred to as SA, SB, SC, and SD and the three loops as LB, LC, and LD.

The 5′CL RNA serves as a platform upon which virus and host proteins gather to initiate replication of the RNA genome [12,13,14]. This region was deemed a cloverleaf because of secondary structure predictions (see Figure 2) that contain one stem without a loop (SA) and three stem-loops (SLB, SLC, and SLD). SLB contains a C-rich loop (see Figure 2) that has been shown to bind the host poly-C-binding protein (PCBP2) [15,16]. Similarly, the host poly-A-binding protein (PABP) binds to the 3′ genomic poly-A tail. Affinity between genome-bound PABP and PCBP2 then effectively circularizes the RNA genome, facilitating multiple rounds of RNA replication [17]. SLD has been shown to bind to the 3CD polyprotein via the SLD loop region [18,19]. This interaction brings 3D^pol^ into proximity with both ends of the genome. Subsequently, 3CD is separated into 3C^pro^ and 3D^pol^ by the action of a separate 3C^pro^ molecule [20,21]. This cleavage releases 3D^pol^ to replicate the genome.

Interestingly, host PCBP2 can also bind to a C-rich region within the IRES [18,22,23,24,25]. This interaction is required for translation. However, PCBP2 can be cleaved by 3C^pro^, which converts PCBP2 into a form that does not bind the IRES, while still allowing interaction with the SLB of the 5′-CL. This cleavage event thus effects a switch from translation to replication [16,26,27].

### Enterovirus Classification and Viruses Chosen for this Study

**Figure 3:**
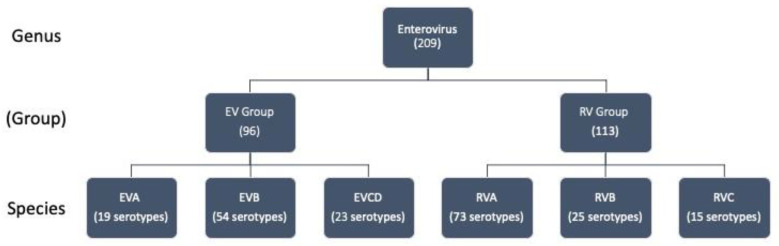
*Enteroviruses* analyzed in this study. The *enterovirus* genus contains both enterovirus and rhinovirus species. For clarity, we have added a group classification (EV group and RV group) to distinguish these species types. This study focuses upon the seven species that are known to infect humans. These include EVA, EVB, and EVCD, in which we have combined the small number of EVC and EVD species, along with RVA, RVB, and RVC. The number of sequences in each category that were used in this analysis is shown in parentheses.

The classification of viruses in this article follows the guidelines of the International Committee for the Taxonomy of Viruses (ICTV), which categorize the serotypes within the *enterovirus* genus according to their genome organization, the amino acid sequence within the polyprotein, and various other factors [2,28,29]. The *enterovirus* genus consists of 15 species. Paradoxically, this genus includes not only 12 enterovirus species (EV A, B, C, D, E, F, G, H, I, J, K, and L) but also three rhinovirus species (RV A, B, and C). *Enteroviruses* known to infect humans fall primarily into one of seven species, EV A–D and RV A–C [28]. Therefore, the current analysis focuses upon these seven species (see Figure 3). For clarity, we use the term “*enterovirus*” to denote the genus and refer to the species groups as the EV group (containing species EVA, EVB, EVC, and EVD) and the RV group (containing species RVA, RVB, and RVC). Due to the limited availability (six serotypes) of complete EVD 5′CL sequences, the EVD sequences have been pooled with the largely similar EVC sequences (34 serotypes) to create a single effective species (EVCD) for statistical analysis. In total, we compared the 5′CL sequence of 209 serotypes. The number of serotypes that fall within each group and each species is indicated in Figure 3.

Antiviral therapeutic development for *enteroviruses* is challenging due, in part, to the high mutation rate for single-stranded RNA viruses, along with the usual challenges of potential side effects and time/cost issues associated with FDA approval [30,31,32]. A potential broad-spectrum antiviral could alleviate some of these issues, particularly as the cost would be balanced vs. the benefit of treating a wide range of ailments. Ribavirin is one example of a broad-spectrum inhibitor of RNA viruses with some efficacy. However, the potency of Ribavirin is not uniform across all RNA viruses. In fact, picornavirus replication is nearly unaffected by Ribavirin [33,34]. While the mechanism of Ribavirin inhibition is not fully understood, one potential means of viral inhibition is through competitive binding at known protein binding sites in the RNA genome. Hagey and associates have explored the potential of this method of viral targeting for influenza A and SARS-CoV-2 through a process they call “programmable antivirals” [35]. In this method, the conservation of both the sequence and secondary structure are explored for regions of high conservation prior to the development and testing of antisense oligonucleotides (ASOs). This method demonstrates the potential to design sequence-targeted antivirals for use against multiple viral serotypes which contain evolutionally conserved regions. Due to the high conservation present in the *enterovirus* 5′CL, therapeutics designed to target the 5′CL may possibly be engineered with broad-spectrum antiviral capabilities.

Here, we explore the sequence conservation of the *enterovirus* 5′CL in more detail than has been previously available, in order to better facilitate potential targeting for broad-spectrum antiviral therapeutics, such as antisense oligonucleotides. Our approach was to analyze the composition and conservation of the 5′CL within the *enterovirus* genus (seven species), within the EV group, and within the RV group. We also performed pairwise comparisons between the EV group and the RV group, and between the separate species within each group, to further assess variability within the *enterovirus* 5′-CL. Finally, we re-classified the 5′-CL independent of group and species classifications and identified the presence of six distinct types of 5′CL that do not precisely align with the ICTV classifications of the complete viral genome. These conservations and variations, once understood, will provide further insight into the role of the *enterovirus* 5′-CL in virus replication and provide further avenues for therapeutic targeting of the *enteroviruses*.

## 2. Materials and Methods

### 2.1. Sequence Collection and Processing

While the *enteroviruses* are known to include over 300 virus types, sequence availability limited the number of serotypes included in this study. Initially, 257 sequences, one per serotype, were collected from GenBank using filters for homo sapiens and completeness. Sequence alignments were then produced to determine a complete cloverleaf within the first 100 nucleotides. Any sequence with a missing or incomplete cloverleaf was removed from the analysis. For the remaining 209 sequences, the first 100 nucleotides were extracted and alignments were produced. The following procedure will utilize the extracted sequences unless otherwise stated. Accession numbers for all sequences used in this analysis are provided in Tables S1–S6.

### 2.2. Sequence Alignment

**Figure 4:**
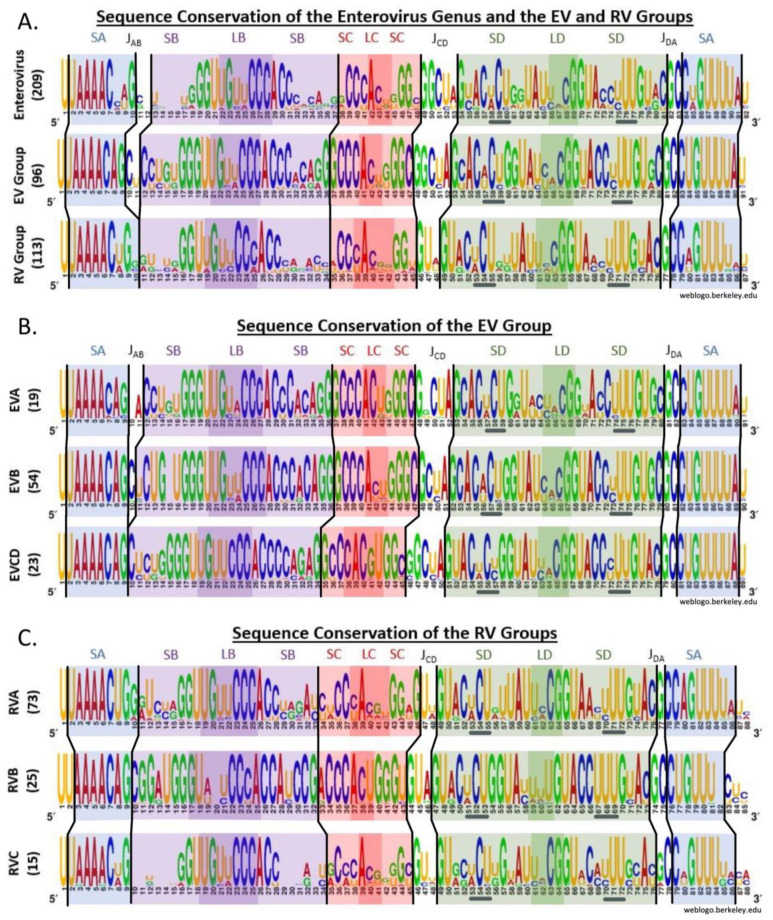
Comparison of 5′CL sequence conservation of the *enterovirus* genus, groups, and species. Sequence conservation (**A**) within the *enterovirus* genus and within the EV and RV groups, (**B**) within each EV species, and (**C**) within each RV species. The height of the stack at each location indicates the conservation level, while the height of each nucleotide indicates its relative frequency. The location within the secondary structure is indicated by the background color, with lighter background shades used for stems and darker shades for the loops. J indicates junction regions.

**Figure 5:**
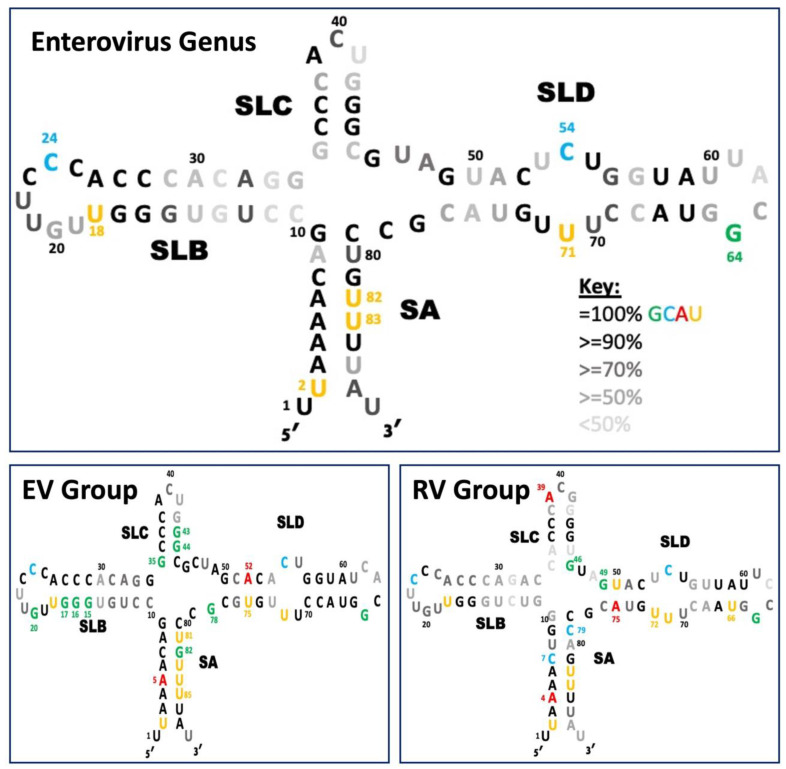
Conservation of the 5′CL sequence. The nucleotides that are both colored and numbered indicate positions that are conserved in the enterovirus genus (**top**), only in the EV group (**bottom left**), and only in the RV group (**bottom right**). The fading gray scale indicates lower levels of conservation (see the inset Key). Black numbering of positions 10, 20, 30, 40, 50, 60, 70, and 80 is for reference only.

We utilized LocARNA software version 1.9.1 to align the 5’CL sequences with folding considerations, which increases alignment accuracy [36,37,38]. However, LocARNA input is limited to 30 sequences. This was of no consequence (see Figure 3) for alignment of EVA (19 serotypes), EVCD (23 serotypes), RVB (25 serotypes), and RVC (15 serotypes). For all other alignments, including EVB (54 serotypes), RVA (73 serotypes), the EV group (96 serotypes), the RV group (113 serotypes), and the *enterovirus* genus (209 serotypes), separate LocARNA results were combined using CLC Main Workbench 22 (QIAGEN, Redwood, CA, USA) (http://www.clcbio.com/, accessed on 30 March 2022) to create accurate alignments of the listed number of serotypes. For each of these more numerous alignments, multiple non-overlapping groups of 30 or fewer sequences were first aligned with LocARNA to cover the required number of serotypes. Next, these partial alignments were transferred into the CLC Main Workbench and combined utilizing the “no redo” function, which maintains each partial alignment but allows them to move relative to one another. The result was an alignment of a large number of sequences with the accuracy of the LocARNA folding-based algorithm applied. Representation of the alignments was completed using Berkley weblogo version 2.8.2 (Figure 4) [39,40]. Analysis of the alignments was conducted to determine positional, sectional, and complete nucleotide compositions. Representation of positional conservation in the genus and in the groups was performed using the respective consensus sequences folded based upon the LocARNA-CLC alignments (Figure 5).

### 2.3. Sequence Similarity

Additional sequence alignments were completed to compare nucleotide distributions on a pair-wise basis.

At each position of the alignment, the nucleotide distribution is represented as a nucleotide vector [G,C,A,U,∅], representing the probabilities of G, C, A, U, and blank occurring at each position of the alignment. Each position *i* represents the location of the nucleotide along the RNA in the alignment. The alignments were used to determine the sequence similarity at each position *i* within the cloverleaf, using the cosine similarity value, which is defined as

$$CosSim\left(X_{i},Y_{i}\right)=\frac{X_{i}}{\left\|X_{i}\right\|}\cdot \frac{Y_{i}}{\left\|Y_{i}\right\|},$$

where *X_i_* and *Y_i_* are the *i*th nucleotide vectors in the alignments of the two sequences, *X* and *Y*, respectively, and ‖∙‖ denotes the Euclidean distance.

### 2.4. Phylogenetic Tree

Utilizing the full-length multiple sequence alignments and the extracted sequence alignments, distance-based phylogenetic trees were generated using the CLC Main Workbench 22 (QIAGEN) (http://www.clcbio.com/ accessed on 1 June 2024). Phylogenetic trees were constructed using neighbor-joining methods [41], while the evolutionary distances were determined using the Jukes–Cantor model [42]. To determine the reliability of the inferred tree, a bootstrap test was conducted using 1000 replicates. Alternative phylogenetic analyses using a neighbor-net algorithm were also performed using SplitTree4 [43,44].

## 3. Results

### 3.1. Phylogenetic Analysis

**Figure 6:**
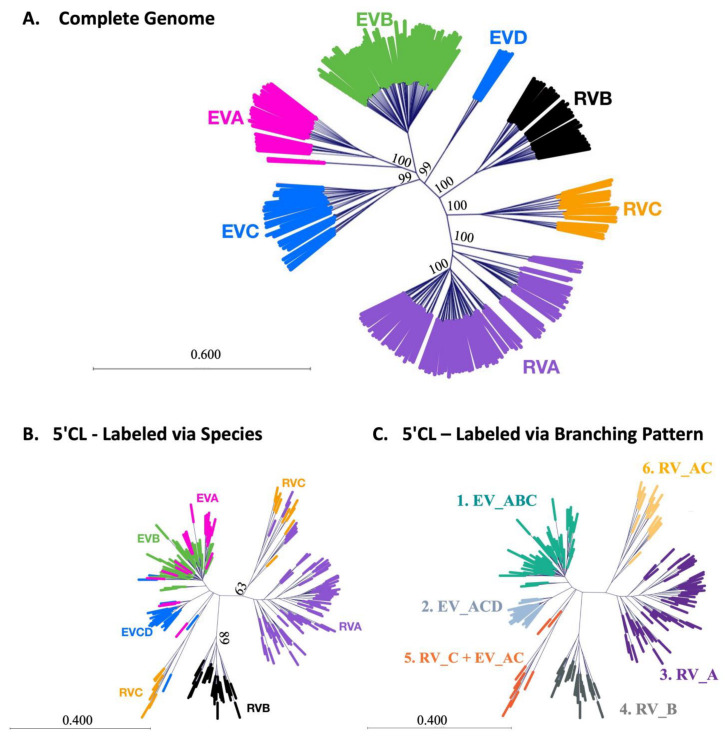
Phylogenetic relationships of (**A**) the whole genome and (**B**,**C**) the 5′CL for the 209 enterovirus sequences in this study. Coloring is provided for clarity. In parts A and B, coloring is used to distinguish species. In part C, coloring is used to distinguish the major phylogenetic branches that emerge from the analysis. Each of the six major branches was then named according to the species that contribute to the branch. This effectively identifies six distinct types of 5′CL that do not align precisely with group and species classifications. Phylogenetic analysis was performed via the neighbor-joining method. Evolutionary distances were computed using the Jukes–Cantor method. The percent bootstrap values, out of 1000 replicates, are shown for the main nodes.

Phylogenetic analysis of the complete RNA genome (~7500 nts) using the neighbor-joining method demonstrates the relationship between the species of the *enterovirus* genus (Figure 6A). First, note that the EV species cluster together (top left) and the RV species cluster together (bottom right). Within the EV species, the branch points (where EVA, EVB, and EVCD diverge) are tightly clustered, indicating high similarity. The RV branch points (where RVA, RVB, and RVC diverge) are more distant from each other, indicating more variability within the RV group. Within the RV group, RVC is phylogenetically intermediate between RVA and RVB. RVA displays high internal variation, as evidenced by multiple branch points within the RVA group. The closest point of approach between the EV and RV groups is between EVCD and RVB. An alternative analysis using the neighbor-net method gives similar results (see Figure S1A).

In contrast, phylogenetic analysis of the 5′CL (Figure 6B) using the neighbor-joining method shows incomplete separation of species. While RVB separates well from other species, EVA overlaps considerably with EVB and, to a lesser extent, with EVCD. Two disparate branches of RVC overlap with minor branches of RVA and of EVCD. The large vertical separation between the RVB branch and the RVA/RVC branch effectively divides the 5′CL sequences into two major groups. This division is made even more clear in the neighbor-net analysis (see Figure S1B).

To facilitate the analysis of the key differences between the 5′CL for each branching cluster in Figure 6B, the serotypes were divided based on branching (see Table S7) and new branch labels were created (see Figure 6C). These six labels identify six distinct forms of the 5′CL from the *enterovirus* genus. The specific areas of conservation and variability affecting these classifications will be shown in the Section 4, along with a discussion of the role they may play in potential virus targeting.

### 3.2. Sequence Composition

The 209 *enterovirus* sequences were first analyzed for simple nucleotide composition (see Figures S2 and S3). Watson–Crick base pairing generally drives composition towards equivalence of G and C and equivalence of A and U. However, non-canonical base pairing and non-base paired loop and bulge regions can drive the composition away from this equivalence. This is the case for the *enterovirus* genus which, as a whole, shows high U content and low A content (GCAU = 23:26:19:29%). The pattern is similar for the RV group, with even higher U content (GCAU = 22:24:21:32%). However, the EV group is rich in C and poor in A (GCAU = 25:29:18:25%). Thus, the RV group substitutes excess C for excess U, but both groups are pyrimidine rich: the EV group contains 56% C and U, while the RV group contains 54% C and U.

The stem regions of the enterovirus display a relatively balanced composition of G to C and A to U, with a slight preference for G and U due to non-conical base pairing such as G•U pairs (GCAU = 25:23:24:27%). As expected, less balance is seen within the loop and bulge regions, in which pyrimidines dominate (GCAU = 19:32:11:33%). Notably, the C-rich loop of SLB contributes to high C content, while the predicted bulge in SLD contributes to high U content.

### 3.3. Sequence Conservation

Sequence logo analysis (Figure 4) uses a multiple sequence alignment to provide information on the consensus sequence and the frequency of nucleotide occurrence at each position within the sequence. At each position, a stack of nucleotides is present: the stack height correlates with the sequence conservation level, while the height of each nucleotide within the stack indicates its relative frequency.

#### 3.3.1. *Enterovirus* Genus Conserved Regions Include SA and Loop B

In the *enterovirus* genus (Figure 4A, first panel), one of the most prominent areas of conservation is SA, presumably related to the required binding of VPg [45]. Additionally, high conservation is exhibited in the cytosine-rich loop B (bases 26–28) and adjacent base pairs (G19•C31, G20•C30, and U21•A29), presumably related to the required PCBP2 interaction [15,16].

In contrast, SLD exhibits low conservation levels in the loop where 3CD is known to bind, other than G69 at the 3′ end of the loop. Nearby base pairs are highly conserved, as is the predicted SLD 3 × 3 bulge (the underlined bases 58–60 and 75–77 in Figure 4A), particularly the central bases C59 and U76, along with U77.

Two C•G base pairs are highly conserved in the SLC stem. However, the most highly conserved position in SLC is A42, the first loop nucleotide. Additionally, the J_CD_ (49–53) and J_DA_ (82–83) junction regions between stem-loops are highly conserved.

#### 3.3.2. EV Is more Highly Conserved than RV, due to Stems, and Has More Junction Bases

Overall, the EV group (Figure 4A, second panel) demonstrates higher conservation than the RV group (Figure 4A, third panel). The extra conservation is mostly in stems B, C, and D. In addition, SA is one base pair shorter in the EV group vs. the RV group. This creates a small one-to-two nucleotide junction region between SA and SLB (J_AB_) in the EV group. The EV group also has larger J_CD_ and J_DA_ regions than the RV group. Thus, in total, the EV group has nine junction bases, while the RV group has only four junction bases.

#### 3.3.3. EVB Is Highly Conserved; EVCD Has a Unique SLC and Lacks J_AB_

Among the EV species (Figure 4B), EVB is unique in its very high conservation throughout stem B, apart from base 15, and in having a smaller J_CD_ region. EVCD is unique in having a shortened SLC stem which lengthens loop C to five bases and in lacking a J_AB_ region. These unique characteristics of EVCD are both in common with the RVA species (see below).

#### 3.3.4. RVB Is Highly Conserved; RVC Is Poorly Conserved

RVB is the most highly conserved RV species, RVC is very poorly conserved, and RVA is intermediate in conservation (Figure 4C). The additional RVB conservation lies mainly in stem B and in SLC. Surprisingly, the first half of RVB loop B is poorly conserved. RVC shows very little conservation in the SLB stem, as well as in SLC. The high RVC variation and, to a lesser extent, the RVA variation are responsible for the lower conservation of the RV group relative to the EV group that was discussed above.

The high overall RVB conservation is similar to that of the EV species: only the loops of SLB and SLD show high variation. RVB also has the smallest 5′CL (80 nts). The overall length of the 5′CL from RVA and RVC is 85 nucleotides, which is intermediate between the lengths of RVB (80 nts) and the EV species (87–89 nts).

### 3.4. Consensus Sequence of Each Species

**Figure 7:**
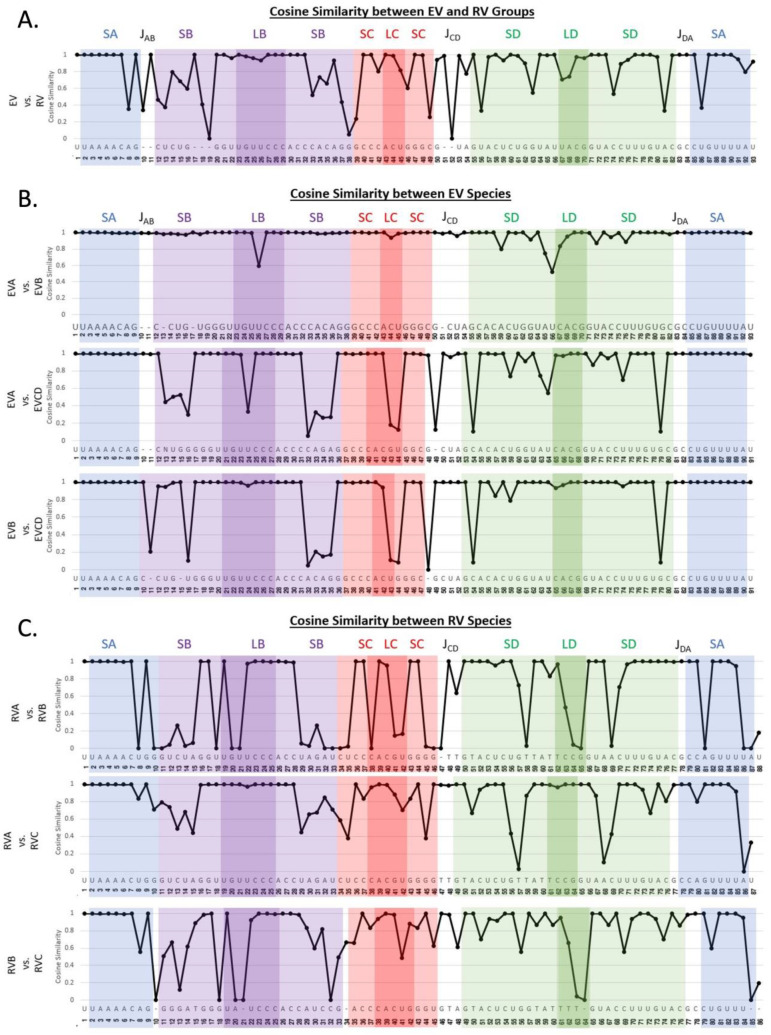
Pairwise cosine similarity of the 5′CL sequences. Pairwise comparison was performed between (**A**) the EV group and the RV group, (**B**) EV species, and (**C**) RV species. A value of 1.0 indicates perfect similarity at that position, while a value of 0.0 indicates complete divergence. The color scheme is as in Figure 4.

**Figure 8:**
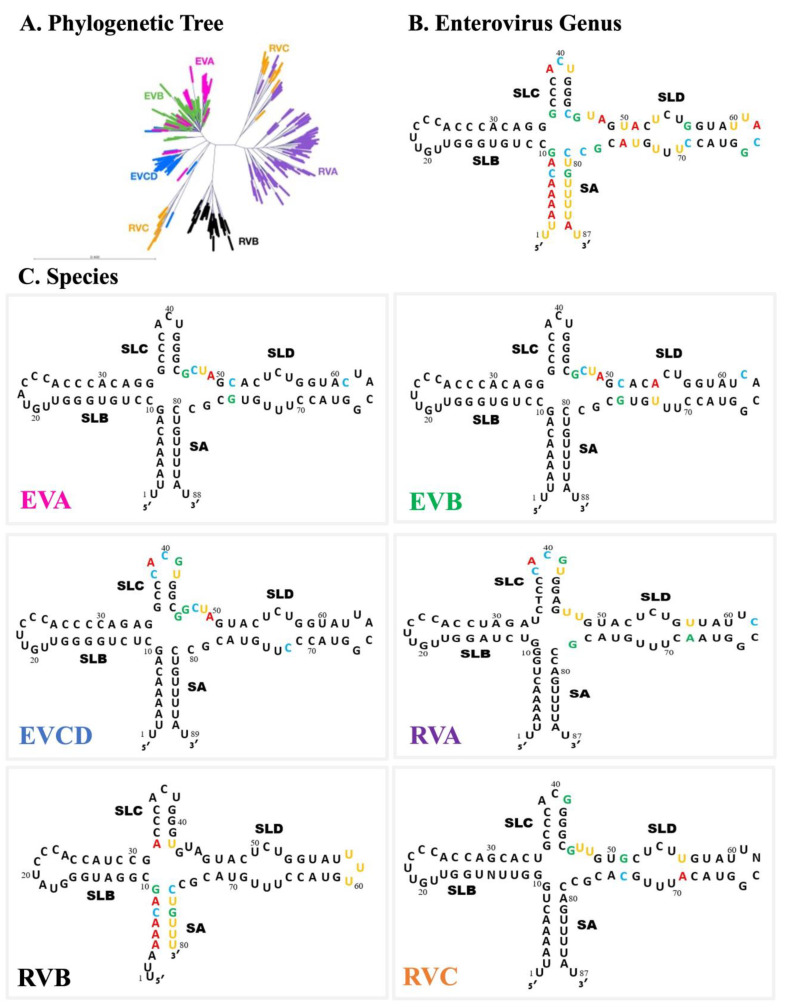
5′CL consensus sequence for the *enterovirus* genus and for each species. (**A**) The 5’CL phylogenetic tree from Figure 6B (for reference). (**B**) The 5′CL consensus sequence for the *enterovirus* genus. The positions with the highest variability are colored (GCAU). (**C**) The 5’CL consensus sequence for each species. Positions with high divergence from the genus consensus sequence of part B are colored (GCAU).

Cosine similarity analysis (Figure 7) presents a pairwise comparison between the GCAU distribution at each position. For instance, in Figure 7A, the value of 1 at positions 1–7 indicates identical GCAU distributions at these positions for the EV group vs. the RV group. The positions need not be invariant to have identical distributions. The results are detailed below and can be used to identify the consensus sequence of each species, as illustrated in Figure 8.

- EV vs. RV
  ○ Similar: SA and loop B and surrounding stem region
  ○ Differ: a single base pair in SA, stem B away from the loop, two base pairs in stem D, and point variations in J_AB_ and J_CD_
- EVA vs. EVB
  ○ Similar: throughout
  ○ Differ: two major divergent bases occur in loop B and near loop D
- EVA/EVB vs. EVCD
  ○ Similar: throughout SA and the loop and surrounding bases of SLB
  ○ Differ: the stems of SLB and SLD as well as two positions (42–43) in SLC
- RVA vs. RVB
  ○ Similar: SA and most of the stem of SLD
  ○ Differ: the stem of SLB adjacent to the junction, in SLC, and various nucleotides in SLD
- RVA vs. RVC
  ○ Similar: nearly identical in loop B and D, along with a majority of SA
  ○ Differ: the stem regions of SLB, SLC, and SLD
- RVB vs. RVC
  ○ Similar: SA and part of SLB near the PCBP2 binding location
  ○ Differ: the stem of SLB and throughout the SLC and SLD

## 4. Discussion

Development of a broad-spectrum antiviral relies on the ability to target an area of high evolutionary conservation. It has previously been established that the *enterovirus* genus 5’CL is highly conserved [46,47]. Nonetheless, this simple statement is an over-simplification. For instance, an antiviral designed to target the 5′CL of RVC might be expected to be effective against the majority of serotypes within the species, if not the majority of the genus. However, as shown in our 5′CL phylogenetic analysis (see Figure 6B), RVC separates into two distinct clusters, one lying between EVCD and RVB species and the other overlapping a minor branch of RVA. Therefore, a designed antiviral may only be effective against a select group of serotypes in the species. The analysis presented here provides a deeper understanding of potential antiviral targets within the 5′CL sequence. This information can be used to target the broadest spectrum of *enterovirus* serotypes, or alternatively, to more comprehensively predict the serotypes expected to be targeted by a specific antiviral.

**Figure 9:**
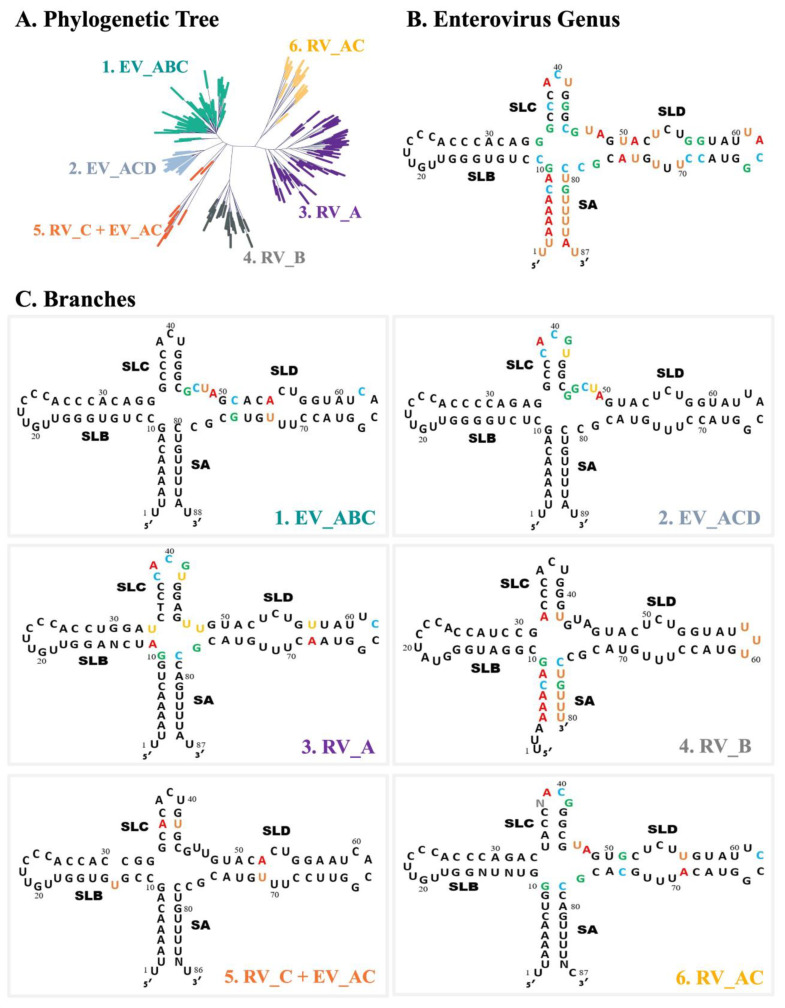
5′CL consensus sequence for the *enterovirus* genus and for each 5′CL phylogenetic branch. (**A**) The 5’CL phylogenetic tree from Figure 6C (for reference). (**B**) The 5′CL consensus sequence for the *enterovirus* genus. The positions with the highest variability are colored (GCAU). (**C**) The 5’CL consensus sequence for each major branch in part A. Positions with high divergence from the genus consensus sequence of part B are colored (GCAU).

Figure 8 presents the 5′CL consensus sequence for each species in this study and highlights the major differences between the species, as explained in the results. These distinct differences limit potential broad-spectrum antiviral targets of the 5′CL sequence due to the need for a consecutive span of conserved residues. However, the cloverleaf phylogeny (Figure 6B) does not directly parallel that of the whole genome (Figure 4A). In fact, the branching pattern of Figure 6B suggests six distinct cloverleaf varieties, some of which contain representatives from more than one species. The sequence characteristics of these six cloverleaves are described below and are illustrated in Figure 9.

1. EV_ABC: a four-nucleotide J_CD_, C51•G76 in SD, A54•U73 base pair which reduces the SD bulge to 2 × 2, and C62 in LD
2. EV_ACD: a five-nucleotide LC and an additional G in J_CD_
3. RV_A: A11•U33 in SB, a five-nucleotide LC, U57•A68 in SD, C62 in LD, a two-nucleotide (UU) J_CD_, and one-nucleotide (G) J_DA_
4. RV_B: short SA (6 bp), A32•U42 in SC, and a three-nucleotide LD
5. RV_C + EV_AC: a predicted one-nucleotide bulge in SB (U13), A36•U42 in SC, and A52•U71 which reduces the SD bulge to 2 × 2
6. RV_AC: a four-nucleotide LC, G51•C74 and U56•A69 in SD, C62 in LD, and short junction regions (J_CD_:2, J_DA_:1)

A complete list of the viruses assigned to each of the six categories above is provided as Supplementary Table S7.

### 4.1. Relating Protein Interactions to Sequence Conservation

While secondary structure predictions of the 5′CL from the *enterovirus* are largely conserved, considerable sequence variation is seen, as presented above. Only eight nucleotide positions are 100% invariant in the 209 sequences we examined. These positions are U2, U18, C24, C54 G64, U71, U82, and U83. As seen in Figure 5 (colored positions in the top diagram), three of these invariant positions are in SA, two in SLB, and three in SLD. Five of these are U, two are C, and one is G; seven of the eight invariant positions are pyrimidines. Most of these invariant positions are near to or part of known protein binding sites, as discussed below.

#### 4.1.1. Stem A: VPg Binding Site

Stem A is highly conserved in most positions (Figure 5), including the three invariant U nucleotides mentioned above. These three U are involved in highly conserved U•A or A•U base pairs. The U2•A86 pair is lost in RVB and in a few cases for other species, but U2 itself remains. This is likely related to the fact that while U1 is thought to be the site that is bound by VPg to initiate replication, if U1 is missing, then U2 can substitute. The VPg interaction is a covalent bond between the 5′-phosphate of the 5′-most U in the genome and the O4 ring oxygen of the VPg tyrosine [48]. The A5•U83 and A6•U82 base pairs are modified to U•U pairs in a small number of cases, conserving U-containing base pairs at these positions. The necessity for involvement of U in these base pairs may be related in some way to the VPg uridylation process [45]. While SA has a high conservation level, its two base pairing strands are sequentially separated by 65 nucleotides, making it a more challenging target for antivirals that typically are designed to bind to a contiguous sequence. The high conservation level seen at the 5′ end of the cloverleaf genome spans approximately ten nucleotides, while a typical ASO target is between 15–25 nucleotides in length [49].

#### 4.1.2. SLB: PCBP Binding Site

Host PCBP2 is known to interact with the C-rich loop of SLB, which is consistent with the high conservation level of C23 (99%), C24 (100%), and C25 (98%), although only C24 is completely invariant (Figure 5). An invariant center C in this CCC triplet guarantees at least two consecutive C, provided that C23 and C25 are not both substituted in the same serotype. The binding mode of PCBP2 involves two consecutive C [50], so a binding motif is conserved, although the two positions may be C23 and C24 or C24 and C25.

Interestingly, an additional highly conserved CC diad is present in the stem of SLB, just 3′ to the CCC loop region. In fact, in the EV group (Figure 5, bottom left), this stem region contains a CCC motif (nucleotides 27–29). This motif may help to attract PCBP2, or even serve as an alternative PCBP2 binding site, under conditions that involve the opening of these base pairs. For instance, during replication, PCBP2 must be displaced to allow loop B to replicate. Rather than fully releasing the RNA, PCBP2 may shift to this secondary CCC site, which is made available by the unwinding of the stem in preparation for continued replication. There, PCBP2 may await an opportunity to return to its original site. If this is indeed the purpose of the additional CCC motif, then a third highly conserved CCC motif present in SLC (nucleotides 36–38) may play a similar role. Note that in each of these three CCC motifs, at least two consecutive C are always present, as required by PCPB2. This has the potential to be problematic if a designed antiviral is targeting the C-rich region of SLB in the hopes of blocking the PCBP. If the hypothesized purpose of the C-rich region in either the stem of SLB or SLC is correct, then the PCBP may be able to bypass a bound antiviral and circularize the genome through another C-rich site.

Note that two additional C-rich regions, one in the IRES [51] and one between the 5′CL and the IRES [52], have previously been identified as PCBP2 binding sites. These two sites are not base paired in secondary structure predictions, and so presumably do not require unfolding to attract PCBP2. Interaction of PCBP2 with the IRES occurs in the loop/bulge regions of SLA and SLB in Domain IV, where it facilitates efficient translation. The C-rich region between the 5′CL and the IRES also contains two highly conserved CCC motifs that have been found to be essential for replication.

The reason for conservation of U18 is less clear, particularly since base pairing between U18 and A26 is highly, but not completely, conserved. Loss of this base pair is most prevalent in RVB, which typically has a predicted nine-base loop, while other RV species and the EV species typically contains a seven-base loop. This change in loop size could allow RVB to evade a broad-spectrum antiviral designed to interact with SLB of the remaining serotypes. It is possible that U18 participates in some way with positioning or attracting PCBP2. In the SLB stem region further from the loop, base pairing is conserved, but compensatory substitutions can be present. This suggests a structural rather than recognition role for the junction-adjacent region of the SLB stem.

As previously discussed, SLB plays a critical role in replication through the binding of PCBP, making it an ideal target. However, compensatory substitutions in SLB present challenges to the design of an antiviral to target this region. For example, for all species except RVB, a span of 13 conserved nucleotides that includes the PCBP2-binding loop provides a potential target site (see Figure 9C). However, 16 nucleotides is considered the optimal length for stability and specificity of an ASO [32]. Compensatory substitutions in the five SLB base pairs closest to the junction (Figure 5, top section) is the factor that limits conservation to 13 nucleotides. Thus, this region may or may not prove to be an effective target for a broad-spectrum antiviral.

#### 4.1.3. SLC: An Orphan

SLC has no invariant positions and no established protein interactions, making it an unlikely target for antiviral targeting. However, two CG base pairs are highly conserved (part of the CCC motif discussed above). In addition, A39 is nearly invariant (Figure 5), being replaced by a C only in EVC118. The reason for this near-invariance is not clear at this time. Since SLC is short, geometric considerations may allow A39 to base pair with an unpaired U elsewhere in the cloverleaf. One potential location of interaction for A39 is the U-rich bulge of SLD as modeled for the 5′CL Coxsackievirus B3 [46]. This model utilized a novel approach to the crystallization of a 5′CL RNA which had been mutated to attract an antibody fragment and to improve crystallization conditions. It remains to be seen whether this interaction is conserved in other species and in the absence of mutations and crystallization.

#### 4.1.4. SLD Loop: 3CD Binding Site

The 3CD polyprotein is known to interact, via its 3C^pro^ section, with the loop of SLD. This interaction brings the 3D^pol^ region into position to begin replication, which proceeds once 3C^pro^ and 3D^pol^ have been separated through the action of a distinct 3C^pro^ molecule. Surprisingly, this known binding location for 3CD does not show the same level of conservation as that of the PCBP2 from SLB, with only the G64 position invariant (Figure 5).

Most *enterovirus* loop D sequences can be partitioned into either the CNCG (most common in EV group) or UNCG (most common in RV group) motifs known for binding the 3C^pro^ [53,54]. Interestingly, RVB contains a tri-loop in place of the D tetra-loop. It has also been shown that 3C^pro^ from an RVB species can accommodate the D tetraloop from other species [55,56], but 3C^pro^ from other species cannot accommodate the RVB species D tri-loop [19,55,57,58]. This would seem counterintuitive as a tetraloop is obviously larger than a tri-loop. However, the CNCG and UNCG tetraloop motif includes base-pairing, although non-canonical, between positions 1 and 4. Thus, the tetraloop could be viewed as a bi-loop, for which there is ample room in the RVB 3C^pro^ binding pocket. In contrast, the RVB tri-loop may not fit in the 3C^pro^ pocket of other species that are designed to accommodate a “bi-loop”. The invariance of G64 would thus seem to be necessary for the formation of the non-canonical C•G or U•G base pair between positions 1 and 4 of the tetraloop. This interpretation is supported by the fact that the analogous G64 position in the RVB species is not part of the tri-loop. In the NMR-based structure of SLD from RVB14, the analogous G64 base pairs with C60 to close the stem around the UAU tri-loop [59]. This closing base pair was reported to be somewhat distorted, which is consistent with the non-canonical base pair within the tetraloop discussed above. The reason that this distorted closing base pair must include a G at position 64 is not known at this time.

The relatively low conservation of SLD would suggest that this is not an ideal site for targeting with a broad-spectrum antiviral. However, the high conservation seen in the stem adjacent to the loop for the EV group (see Figure 5, bottom left) demonstrates broad-spectrum potential on a narrower scale. Variability in the EV group SLD tetraloop connecting these two stem sequences presents another challenge. However, note that the first position of the SLD loop is almost always either a U or C, each of which can be targeted by a G nucleotide, to form a canonical G•C or non-conical G•U base pair.

#### 4.1.5. SLD 3 × 3 Pyrimidine Mismatch: A Stem Distortion

The predicted 3 × 3 pyrimidine bulge in SLD (positions 53–55 and 70–72) is highly conserved. The central base on each side of the bulge (C54 and U71) is invariant. In addition, the subsequent bases (U55 and U72) are 92% and 99% conserved, respectively. The remaining two bulge positions are less well conserved: in the EV group, U53 is commonly replaced by A, creating a A53•U72 base pair, which reduces the predicted bulge to 2 × 2 (Figure 5, lower left panel); U70 is replaced by C in approximately 30% of the cases, maintaining the predicted bulge at the U55 and U70 position. However, in three model systems, these six nucleotides were shown to form three pyrimidine–pyrimidine base pairs. Therefore, SLD should be considered to contain one long continuous stem [54,59,60]. It is thought that these atypical base pairs distort the helix, resulting in a widened major groove that could be a point of access to host or virus proteins. This would be consistent with the high conservation of these pyrimidine “mismatches”, even if they do not form a bulge.

In recent crystal structures of the 5’CL from CVB3 and poliovirus [46,61], an interaction has been noted between LC and the SD 3 × 3 bulge. Specifically, A39 from LC forms a base triple (A•C-U) with two pyrimidines from the SLD bulge. It was proposed that this interaction helps to position the PCBP and 3CD binding sites (in LB and LD, respectively) on opposite ends of the structure. The generality of this base triple is not yet established beyond CVB3 and poliovirus. However, this could help explain why the A39 position is conserved in 208 of the 209 serotypes in this study. It should also be noted that effective crystallization in these studies required either co-crystallization with tRNA [61] or with an antibody fragment [46] in addition to loop mutations favoring co-crystallization. A previous NMR/small angle X-ray scattering analysis of the RV14 5′CL [62] did not report this contact, but the approach used in this latter study would not have detected the base triple.

#### 4.1.6. Junction Regions: Conserved for a Reason?

In the *enterovirus* genus, five unpaired bases are predicted at the junctions between SLC and SLD (J_CD_; GUA) and between SLD and SA (J_DA_; GC) (Figure 5, top panel). None of these positions are invariant, in part because of larger predicted junction regions in the EV group vs. the RV group: the EV group junctions contain six unpaired bases (J_CD_; GCUA) (J_DA_; GC), while the RV group junctions contain only three unpaired bases (J_CD_; UA) (J_DA_; G).

The larger EV group junctions (Figure 5, bottom left) may instead form two additional base pairs between G46•C79 and C47•G78, as was found in an NMR study of an isolated consensus SLD sequence [54]. These positions are each at least 93% conserved within the EV group. Thus, the actual number of unpaired junction bases may be two for the EV group (J_CD_; UA).

Furthermore, the RV group junction could form an additional U47•G77 base pair which is 96% conserved, leaving only A48 unpaired and unconserved (50%). However, this U•G pair was not detected in an NMR/SAXS analysis of the RVB14 5′CL [62].

The role of these base pairs or unpaired bases is not clear, as they are unlikely to contact proteins. However, they could influence the length of the stems, and hence the relative positions of the terminal loops, as well as the tertiary folding in the form of stacking of the stem regions of SA, SLB, SLC, and SLD. Sequence conservation would not seem to be required for these functions, and yet conservation is high at these positions. It should be borne in mind, though, that bases at the junction region would be surrounded by a large number of potential interacting bases that might produce alternate conformations. Thus, correct stem length and correct stem stacking may rely on the identity of the junction bases.

Regardless of their evolutionary purpose, conserved junction positions can be helpful in targeting by ASOs. For example, the highly conserved GC found in J_DA_ (see Figure 5, top section) provides an additional two conserved nucleotides at the 3′ end of SA.

### 4.2. EV Group Contains 14 and RV Group Contains 10 Additional Invariant Positions

In addition to the 8 positions (discussed above) that are invariant throughout the genus, another 14 positions are invariant only within the EV group (Figure 5, lower left, colored and numbered positions). Nine of these invariant positions are G (GCAU = 9:0:2:3 nucleotides). Seven of these invariant G nucleotides are clustered near LB and SC. As discussed above, the clustering of invariant positions near LB and its role in viral replication makes SLB a promising site for antiviral targeting.

In contrast, only 10 additional positions are invariant only within the RV group (Figure 5, lower right), the nucleotide distribution is random (GCAU = 2:2:3:3), and the positions are less clustered. However, half of these 10 positions fall within SD.

**Figure 10:**
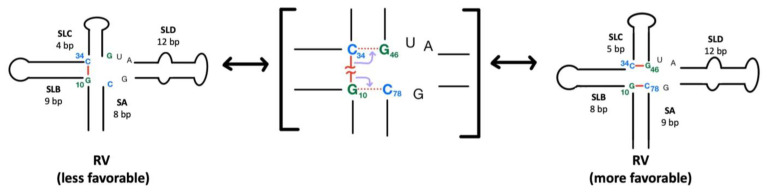
Alternative secondary structures of the RV 5′CL. The G10•C34 base pair (left) is predicted to split in order to form the G10•C78 and C34•G46 base pairs. The result is a shorter SB and longer SA and SC.

Interestingly, inversion of the C10•G34 base pair (in the EV group) to G10•C34 (in the RV group, mostly RVA) changes the length predictions for three of the four stems (see Figure 10). Energetically, the RVA G10•C34 base pair is predicted to split, in favor of formation of G10•C78 and C34•G46 base pairs. The net result is the loss of one base pair in SB and the lengthening of SA and SC by one base pair each. It remains to be seen whether these differences in stem lengths actually occur, but it is intriguing to consider the effect that these compensating adjustments could have on tertiary structure, including on stacking of helices.

### 4.3. Hypervariable Regions

Hypervariable regions provide a major challenge when developing an antiviral to target the 5′CL. The potential to design an ASO to target a conserved region of the 5′CL at a site of known protein-RNA interaction would be ideal, as it could inhibit viral replication on the broadest scale. However, this may be more problematic than the overall high conservation level would suggest. In the *enterovirus* genus, hypervariable regions are present in all stem and loop regions. Only two positions are hypervariable in SA. High variation in the other three stem regions suggests that significant portions of these stems are important not for specific contacts, but for maintaining shape and position. Lack of conservation in each loop suggests that the size of the loops is important, but the content outside of a small recognition site may be unimportant for recognition. For instance, the SLB loop is maintained as seven bases, with only the CCC highly conserved. The remaining non-conserved bases may be important mainly for introducing flexibility needed for access to the CCC. The importance of a tri-loop vs. tetra-loop in SLD has been discussed above.

### 4.4. 5′CL vs. 3′CL

Conservation at various positions in the 5′CL may be the result of a needed function in the synthesis of either negative-sense or positive-sense RNA strands. The present manuscript considers the role of the 5′CL in the synthesis of the negative-sense strand. However, it will also be important to consider the role that invariant positions may play in the synthesis of the positive-sense strand.

In replication, the positive-sense genomic strand is used as a template to make negative-sense RNA strands. The negative-sense strands then serve as a template for the production of additional positive-sense strands. At the 3′-end of the negative-sense strand, a cloverleaf may form from the bases that are complementary to the 5′CL. This is called the 3′CL. Therefore, it is possible that some of the invariant positions in the 5′CL may be required only for their role in the 3′CL.

As in the 5′CL, the 3′CL is utilized in the circularization of the negative-sense strand to increase replication efficiency. This is accomplished via the interaction of heterogeneous nuclear ribonucleoprotein C (hnRNP C) with the 3′CL and the 5′NCR of the negative-sense strand. This interaction may account for additional unexplained invariant positions in the 5′CL, such as in SA, which is thought to interact with hnRNP C [63,64,65]. Indeed, it has been shown in EVB and EVC serotypes that mutation of two of the four consecutive U positions in SA of the 3′CL template strand inhibits the binding of the hnRNP C [66]. This could explain the high conservation observed (≥90%) at the A4 and A5 positions of the 5′CL. Interestingly, A4 is 100% conserved in the RV group, while A5 is 100% conserved in the EV group.

Some invariant positions may play a dual role in both positive and negative strand synthesis. For instance, the two well-conserved U at the 5′-most end of the 5′CL play a role in the uridylation of VPg prior to positive-strand synthesis. The corresponding complementary AA sequence thus would form a short poly-A tail in the 3′CL. This AA diad also binds the uridylated VPg for use as a primer in negative-sense synthesis.

### 4.5. Breadth of Analysis and Comparison with Previous Studies

Previous studies have examined sequence conservation of the *enterovirus* genome. Indeed, the present classification of picornaviruses is based upon sequence analysis, including of the IRES, the ORF, and the CRE (cis-acting replication element) [67,68,69]. Specific analysis of the 5′CL has found high structural (a cloverleaf) and sequence conservation within the *enterovirus* genus [23,47]. A recent analysis used mainly *enterovirus* species (92%), especially EVA and EVB serotypes which we have shown to be nearly identical, to find a consensus sequence [46]. Here, we present an analysis of conserved and variable 5’CL regions within the *enterovirus* genus, which includes both enterovirus and rhinovirus species, including an analysis of variation within and between species and the possible function of these regions in viral replication.

In addition, we took an approach that gave equal weight to each available serotype. Databases show multiple sequence variants for each serotype in our analysis. Rather than include each variant in our study, which would overemphasize those serotypes with many published variants, we chose one variant per serotype for inclusion in our analysis. The designated reference variant was used when available (see About RefSeq (nih.gov)). When a reference variant was not available, the most recently reported variant with a complete 5’CL sequence was used. Since mutation rates are high in single-stranded RNA viruses, this approach of using a recent representative of the serotype for analysis can help to address recent mutational shifts that may affect human health.

Serotypes that did not have full 5’CL deposits in GenBank were excluded from this analysis. Out of 114 existing human-related EV serotypes, 96 sequences were used in this analysis. Out of 172 existing human-related RV serotypes, 113 sequences were used. The majority of serotypes omitted (42 of the 77 omitted species) are from the RVC species category. RVC serotypes are often difficult to culture, many having been identified only via PCR directly on nasal swabs, and thus RVC 5’CL sequence data are relatively scarce [70].

We also limited our analysis to human health-related enteroviruses. This restriction limited the number of EVD serotypes included to five. Thus, to provide statistically meaningful analysis, EVD sequences were grouped with their phylogenetically closest species (EVC), as seen in Figure 4A.

This analysis allowed us to identify six distinct 5′CL forms (Figure 8B).

## 5. Conclusions

The *enterovirus* 5′CL serves as a platform upon which replication of the RNA genome initiates. This platform is known to be highly conserved, but we present here a more comprehensive analysis of the regions of conservation and variability and their distribution over 209 human health-related serotypes of the *enterovirus* genus. Our analysis shows high conservation of the 5′CL secondary structure between species, with RVB as the outlier with a slightly smaller cloverleaf. In addition, sequence conservation is higher between the EV species than between the RV species, with EVA and EVB exhibiting the highest similarity to each other. However, variability is not insignificant. Phylogenic analysis, together with sequence analysis, allowed the definition of six distinct 5′CL isoforms that only partially align with the species classifications. Analysis of invariant or highly conserved positions vs. highly variable regions correlate with and provide additional insight into known functions and interactions and point to new possible interactions or structural requirements that are related to conservation in areas of unknown function, including stem regions from SLB, SLC, and SLD, the SLC loop, and two junction regions. These results afford a more concise analysis of features amenable to targeting via a broad-spectrum antiviral. They also provide for a more accurate prediction of the serotypes expected to be targeted by any particular antiviral.

## Data Availability

The data for this manuscript is computational and will be made available upon request.

## Supplementary Materials

The following supporting information can be downloaded at: https://www.mdpi.com/article/10.3390/v16071009/s1, Table S1: EVA serotypes used in this study (19); Table S2: EVB serotypes used in this study (54); Table S3: EVCD serotypes used in this study (23); Table S4: RVA serotypes used in this study (73); Table S5: RVB serotypes used in this study (25); Table S6: RVC serotypes used in this study (15); Table S7: 5′CL Phylogenetic branches serotype composition; Figure S1: Phylogenetic relationship of (A) the whole genome and (B) 5′CL utilizing a neighbor-net algorithm; Figure S2: Comparison of enterovirus genus, EV group and RV group percent nucleotide composition; Figure S3: Comparison of percent nucleotide composition for each species.
