# Supplementary material for: Exploration of Potential Broad-Spectrum Antiviral Targets in the Enterovirus Replication Element: Identification of Six Distinct 5′ Cloverleaves
Source: Viruses. 2024 Jun 23;16(7):1009. doi: 10.3390/v16071009 (PMC11281424; doi:10.3390/v16071009)
Supplement: Supplementary file 1 [file viruses-16-01009-s001.zip › Figure_S1.pdf]

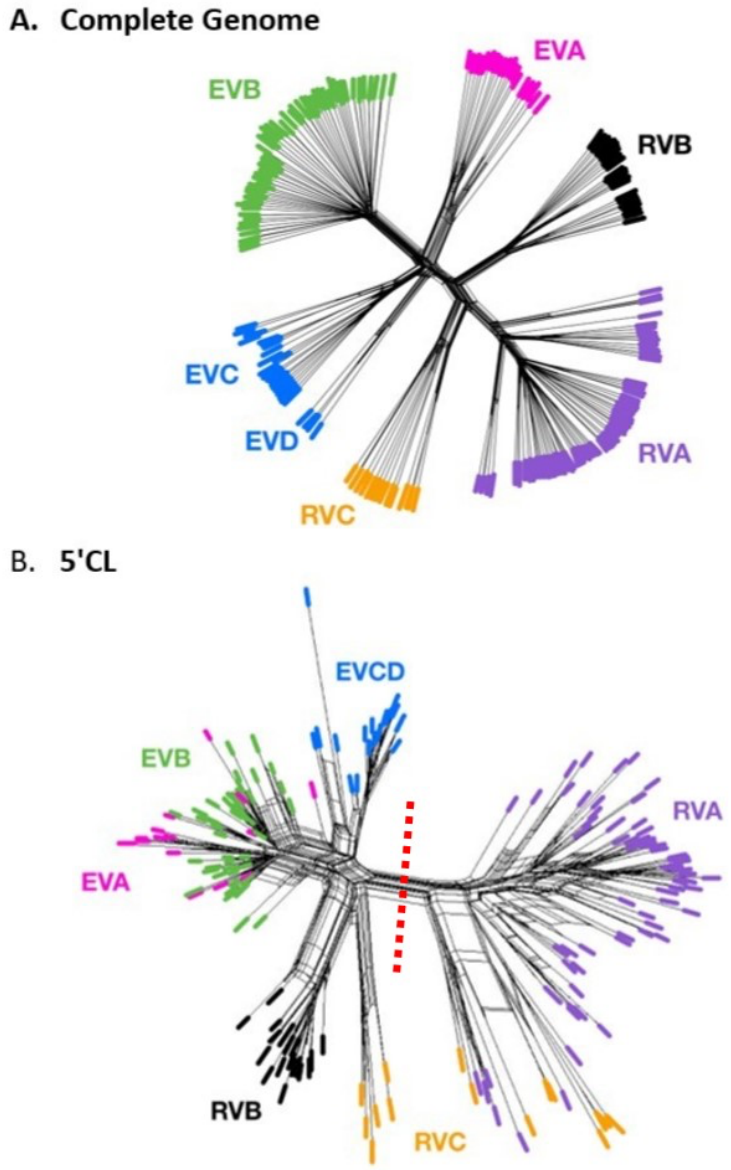

Figure S1: Phylogenetic Relationship (A) the whole genome and (B) 5'CL utilizing a Neighbor-Net Algorithm

Phylogenetic relations of (A) the whole genome exhibit complete separation between the various species within the enterovirus genus, while the (B) 5'CL has substantial overlap. Similar to the neighbor-joining algorithm (Fig. 4), the (B) 5'CL subdivides the sequences into two subgroups as demonstrated by the dotted line. Surprisingly, the two groups are not separated into the rhinoviruses and enteroviruses but instead have RVB and a small portion of RVC grouped with the enterovirus species. However, compared to the neighbor-joining phylogenetic tree (Fig. 4), a shift in the RVB and RVC branching is seen thus moving the small group of RVC closer to both RVB and the RVA/RVC subgroup.

Analysis was via the SplitTree4 software Neighbor-Net method.
