# Supplementary material for: Exploration of Potential Broad-Spectrum Antiviral Targets in the Enterovirus Replication Element: Identification of Six Distinct 5′ Cloverleaves
Source: Viruses. 2024 Jun 23;16(7):1009. doi: 10.3390/v16071009 (PMC11281424; doi:10.3390/v16071009)
Supplement: Supplementary file 1 [file viruses-16-01009-s001.zip › Figure_S2.pdf]

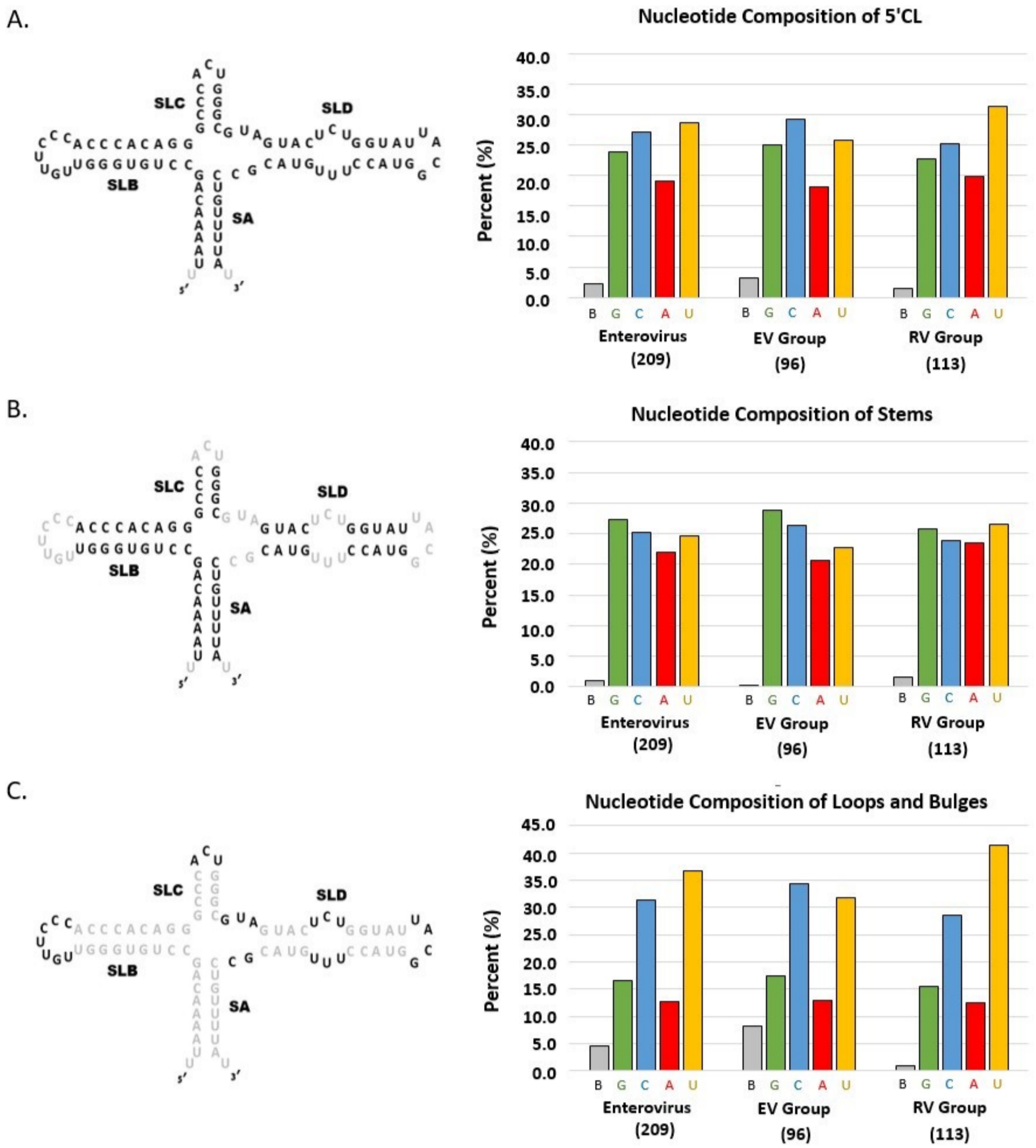

Figure S2: Comparison of Enterovirus genus, EV group and RV group percent nucleotide composition.

Panels analyze (A) the full 5'CL, (B) stems, and (C) loops and bulges. The black nucleotides on the left indicate which positions are analyzed in each panel. The Enterovirus genus contains a higher percentage of C and U in the 5’CL, with the EV group having the highest percentage of C and the RV group having the highest percentage of U. This trend correlates with the nucleotide composition in the loops and bulges. In contrast, in the genus and both groups, A is seen with the lowest percent composition and tracks through both the stems and loops/bulges.
