# Supplementary material for: Exploration of Potential Broad-Spectrum Antiviral Targets in the Enterovirus Replication Element: Identification of Six Distinct 5′ Cloverleaves
Source: Viruses. 2024 Jun 23;16(7):1009. doi: 10.3390/v16071009 (PMC11281424; doi:10.3390/v16071009)
Supplement: Supplementary file 1 [file viruses-16-01009-s001.zip › Figure_S3.pdf]

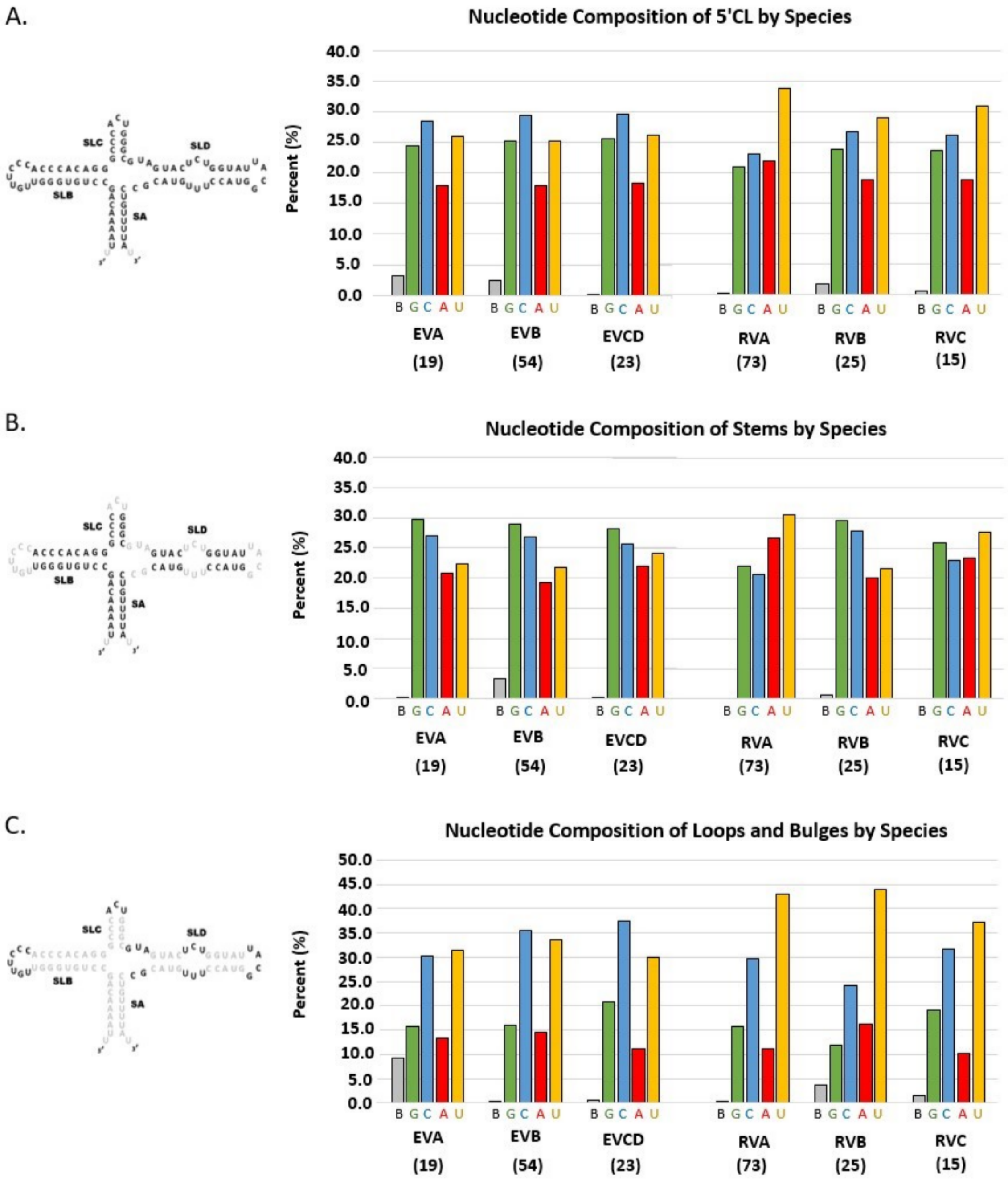

Figure S3: Comparison of percent nucleotide composition for each species.

Panels analyze (A) the full 5'CL, (B) stems, and (C) loops and bulges. The black nucleotides on the left indicate which positions are analyzed in each panel. Interestingly, the EV species (EVA, EVB, EVCD) contain a higher percentage of C with the RV species (RVA, RVB, RVC) having the highest percentage of U, which correlate with the group findings [Figure S2]. This trend correlates with the nucleotide composition in the loops and bulges for each of the species. However, variation in the nucleotide compositions is seen in the stems with G and C being more prevalent in the EV species and RVB.
