# Supplementary material for: Exploration of Potential Broad-Spectrum Antiviral Targets in the Enterovirus Replication Element: Identification of Six Distinct 5′ Cloverleaves
Source: Viruses. 2024 Jun 23;16(7):1009. doi: 10.3390/v16071009 (PMC11281424; doi:10.3390/v16071009)
Supplement: Supplementary file 1 [file viruses-16-01009-s001.zip › Table_S1.pdf]

Table S1: EVA serotypes used in this study (19)

Name	Accession Number	Name	Accession Number	Name	Accession Number
Coxsackievirus A10	MT263729	Coxsackievirus A5	OK334537	Enterovirus A121	KU355877
Coxsackievirus A12	MK061424	Coxsackievirus A6	KR815992	Enterovirus A71	KM077140
Coxsackievirus A14	AY421769	Coxsackievirus A7	MT179786	Enterovirus A76	JF905564
Coxsackievirus A16	MT211988	Coxsackievirus A8	KP289435	Enterovirus A89	KT277550
Coxsackievirus A2	NC_038306	Enterovirus A114	KU355876	Enterovirus A91	AY697461
Coxsackievirus A3	AY421761	Enterovirus A120	MT081367	Enterovirus A92	EF667344
Coxsackievirus A4	MG431822				
