# Supplementary material for: Exploration of Potential Broad-Spectrum Antiviral Targets in the Enterovirus Replication Element: Identification of Six Distinct 5′ Cloverleaves
Source: Viruses. 2024 Jun 23;16(7):1009. doi: 10.3390/v16071009 (PMC11281424; doi:10.3390/v16071009)
Supplement: Supplementary file 1 [file viruses-16-01009-s001.zip › Table_S2.pdf]

Table S2: EVB serotypes used in this study (54)

Name	Accession Number	Name	Accession Number	Name	Accession Number
Coxsackievirus A9	OM177688	Echovirus 21	MN481500	Enterovirus B107	AB426609
Coxsackievirus B1	LN854562	Echovirus 24	AY302548	Enterovirus B69	AY302560
Coxsackievirus B2	EF174469	Echovirus 25	MT347976	Enterovirus B73	KF874626
Coxsackievirus B3	NC_038307	Echovirus 26	AY302550	Enterovirus B74	JQ397329
Coxsackievirus B4	KP289433	Echovirus 27	AY302551	Enterovirus B75	MW183133
Coxsackievirus B5	KF311743	Echovirus 29	MK689070	Enterovirus B77	AY843302
Coxsackievirus B6	KY262575	Echovirus 3	MK791150	Enterovirus B79	AY843297
Echovirus 11	MW883610	Echovirus 30	MW080372	Enterovirus B80	MH614922
Echovirus 12	MF083152	Echovirus 31	AY302554	Enterovirus B81	KJ755189
Echovirus 13	AY302539	Echovirus 33	KT965725	Enterovirus B82	AY843300
Echovirus 14	KP289440	Echovirus 4	FJ172447	Enterovirus B83	MN164683
Echovirus 15	AY302541	Echovirus 5	HM775882	Enterovirus B84	DQ902712
Echovirus 16	KP289436	Echovirus 6	MK791151	Enterovirus B85	JX898905
Echovirus 17	AY302543	Echovirus 7	KP202389	Enterovirus B86	AY843304
Echovirus 18	MN815810	Echovirus 9	KX610685	Enterovirus B87	KC292019
Echovirus 19	MH745407	Enterovirus B100	DQ902713	Enterovirus B93	MN580134
Echovirus 2	AF465518	Enterovirus B101	AY843308	Enterovirus B97	GU550508
Echovirus 20	KX060810	Enterovirus B106	KX171334	Enterovirus B98	AB426608
