# Supplementary material for: Exploration of Potential Broad-Spectrum Antiviral Targets in the Enterovirus Replication Element: Identification of Six Distinct 5′ Cloverleaves
Source: Viruses. 2024 Jun 23;16(7):1009. doi: 10.3390/v16071009 (PMC11281424; doi:10.3390/v16071009)
Supplement: Supplementary file 1 [file viruses-16-01009-s001.zip › Table_S3.pdf]

Table S3: EVCD serotypes used in this study (23)

Name	Accession Number	Name	Accession Number	Name	Accession Number
Coxsackievirus A1	JX174176	Coxsackievirus A24	MK989721	Enterovirus D111	MT081372
Coxsackievirus A11	JF260917	Enterovirus C102	EF555645	Enterovirus D68	NC_038308
Coxsackievirus A13	JF260920	Enterovirus C104	KR815824	Enterovirus D70	MT081369
Coxsackievirus A17	JF260924	Enterovirus C109	GQ865517	Enterovirus D94	MT081370
Coxsackievirus A19	MT175706	Enterovirus C117	MT338567	Poliovirus 1	MH750912
Coxsackievirus A20	DQ358078	Enterovirus C118	JX678288	Poliovirus 2	MZ245455
Coxsackievirus A21	EF015030	Enterovirus C96	MK512666	Poliovirus 3	MK028134
Coxsackievirus A22	JN542510	Enterovirus C99	MK689071		
