# Supplementary material for: Exploration of Potential Broad-Spectrum Antiviral Targets in the Enterovirus Replication Element: Identification of Six Distinct 5′ Cloverleaves
Source: Viruses. 2024 Jun 23;16(7):1009. doi: 10.3390/v16071009 (PMC11281424; doi:10.3390/v16071009)
Supplement: Supplementary file 1 [file viruses-16-01009-s001.zip › Table_S4.pdf]

Table S4: RVA serotypes used in this study (73)

Name	Accession Number	Name	Accession Number	Name	Accession Number
Rhinovirus A1	NC_038311	Rhinovirus A36	DQ473505	Rhinovirus A65	FJ445147
Rhinovirus A10	FJ445178	Rhinovirus A38	FJ445180	Rhinovirus A66	FJ445148
Rhinovirus A100	FJ445175	Rhinovirus A39	AY751783	Rhinovirus A67	FJ445149
Rhinovirus A101	GQ415051	Rhinovirus A40	MK167031	Rhinovirus A68	FJ445150
Rhinovirus A103	JF965515	Rhinovirus A41	MN369037	Rhinovirus A7	FJ445176
Rhinovirus A11	EF173414	Rhinovirus A43	FJ445131	Rhinovirus A71	FJ445152
Rhinovirus A12	EF173415	Rhinovirus A44	DQ473499	Rhinovirus A73	DQ473492
Rhinovirus A13	FJ445117	Rhinovirus A45	MN749144	Rhinovirus A74	DQ473494
Rhinovirus A15	DQ473493	Rhinovirus A46	DQ473506	Rhinovirus A75	DQ473510
Rhinovirus A16	L24917	Rhinovirus A47	FJ445133	Rhinovirus A76	DQ473502
Rhinovirus A18	FJ445118	Rhinovirus A49	FJ445134	Rhinovirus A77	FJ445154
Rhinovirus A19	FJ445119	Rhinovirus A50	FJ445135	Rhinovirus A8	FJ445113
Rhinovirus A1B	D00239	Rhinovirus A51	FJ445136	Rhinovirus A80	FJ445156
Rhinovirus A2	X02316	Rhinovirus A53	DQ473507	Rhinovirus A81	FJ445157
Rhinovirus A20	FJ445120	Rhinovirus A54	FJ445139	Rhinovirus A82	DQ473509
Rhinovirus A21	KR090587	Rhinovirus A56	FJ445140	Rhinovirus A85	OK161391
Rhinovirus A22	FJ445119	Rhinovirus A57	FJ445141	Rhinovirus A88	DQ473504
Rhinovirus A25	MZ153273	Rhinovirus A58	LC699420	Rhinovirus A89	M16248
Rhinovirus A28	MZ670592	Rhinovirus A59	DQ473500	Rhinovirus A9	FJ445114
Rhinovirus A29	OK649361	Rhinovirus A60	FJ445143	Rhinovirus A90	FJ445167
Rhinovirus A30	DQ473512	Rhinovirus A61	OK161354	Rhinovirus A94	FJ445185
Rhinovirus A31	FJ445126	Rhinovirus A62	FJ445145	Rhinovirus A95	FJ445170
Rhinovirus A32	FJ445127	Rhinovirus A63	FJ445146	Rhinovirus A96	FJ445171
Rhinovirus A33	FJ445128	Rhinovirus A64	FJ445181	Rhinovirus A98	FJ445173
Rhinovirus A34	DQ473501				
