# Supplementary material for: Exploration of Potential Broad-Spectrum Antiviral Targets in the Enterovirus Replication Element: Identification of Six Distinct 5′ Cloverleaves
Source: Viruses. 2024 Jun 23;16(7):1009. doi: 10.3390/v16071009 (PMC11281424; doi:10.3390/v16071009)
Supplement: Supplementary file 1 [file viruses-16-01009-s001.zip › Table_S5.pdf]

Table S5: RVB serotypes used in this study (25)

Name	Accession Number	Name	Accession Number	Name	Accession Number
Rhinovirus B14	X01087	Rhinovirus B48	DQ473488	Rhinovirus B83	FJ445161
Rhinovirus B17	EF173420	Rhinovirus B5	FJ445112	Rhinovirus B84	FJ445162
Rhinovirus B26	FJ445124	Rhinovirus B52	FJ445188	Rhinovirus B86	FJ445164
Rhinovirus B27	FJ445186	Rhinovirus B6	DQ473486	Rhinovirus B91	KX494871
Rhinovirus B3	NC_038312	Rhinovirus B69	FJ445151	Rhinovirus B92	FJ445169
Rhinovirus B35	DQ473487	Rhinovirus B70	DQ473489	Rhinovirus B93	EF173425
Rhinovirus B37	EF173423	Rhinovirus B72	FJ445153	Rhinovirus B97	FJ445172
Rhinovirus B4	DQ473490	Rhinovirus B79	MT512399	Rhinovirus B99	FJ445174
Rhinovirus B42	FJ445130				
