# Supplementary material for: Exploration of Potential Broad-Spectrum Antiviral Targets in the Enterovirus Replication Element: Identification of Six Distinct 5′ Cloverleaves
Source: Viruses. 2024 Jun 23;16(7):1009. doi: 10.3390/v16071009 (PMC11281424; doi:10.3390/v16071009)
Supplement: Supplementary file 1 [file viruses-16-01009-s001.zip › Table_S6.pdf]

Table S6: RVC Serotypes used in this study (15)

| Name | Accession Number | Name | Accession Number | Name | Accession Number |
|---|---|---|---|---|---|
| Rhinovirus C10.F | MZ363422 | Rhinovirus C42.F | MH752985 | Rhinovirus C54.F | KP282614 |
| Rhinovirus C15.F | GU219984 | Rhinovirus C43.F | KY369878 | Rhinovirus C55.F | MW969530 |
| Rhinovirus C26.F | MZ363457 | Rhinovirus C5.F | EF582386 | Rhinovirus C6.F | EF582387 |
| Rhinovirus C3.F | EF186077 | Rhinovirus C51.F | JF317015 | Rhinovirus C7.F | OK254850 |
| Rhinovirus C4.F | EF582385 | Rhinovirus C53.F | MF775367 | Rhinovirus C8.F | GQ223227 |
