# Supplementary material for: Exploration of Potential Broad-Spectrum Antiviral Targets in the Enterovirus Replication Element: Identification of Six Distinct 5′ Cloverleaves
Source: Viruses. 2024 Jun 23;16(7):1009. doi: 10.3390/v16071009 (PMC11281424; doi:10.3390/v16071009)
Supplement: Supplementary file 1 [file viruses-16-01009-s001.zip › Table_S7.pdf]

Table S7: 5’CL Phylogenetic Branches Serotypes Composition

**EV_ABC**						
Coxsackievirus A2	Coxsackievirus A3	Coxsackievirus A4	Coxsackievirus A5	Coxsackievirus A6	Coxsackievirus A7	Coxsackievirus A8
Coxsackievirus A9	Coxsackievirus A10	Coxsackievirus A12	Coxsackievirus A14	Coxsackievirus A16	Coxsackievirus B1	Coxsackievirus B2
Coxsackievirus B3	Coxsackievirus B4	Coxsackievirus B5	Coxsackievirus B6	Echovirus 2	Echovirus 3	Echovirus 4
Echovirus 5	Echovirus 6	Echovirus 7	Echovirus 9	Echovirus 11	Echovirus 12	Echovirus 13
Echovirus 14	Echovirus 15	Echovirus 16	Echovirus 17	Echovirus 18	Echovirus 19	Echovirus 20
Echovirus 21	Echovirus 24	Echovirus 25	Echovirus 26	Echovirus 27	Echovirus 29	Echovirus 30
Echovirus 31	Echovirus 33	Enterovirus A71	Enterovirus A76	Enterovirus A89	Enterovirus A92	Enterovirus A114
Enterovirus A120	Enterovirus B69	Enterovirus B73	Enterovirus B74	Enterovirus B75	Enterovirus B77	Enterovirus B79
Enterovirus B80	Enterovirus B81	Enterovirus B82	Enterovirus B83	Enterovirus B84	Enterovirus B85	Enterovirus B86
Enterovirus B87	Enterovirus B93	Enterovirus B97	Enterovirus B98	Enterovirus B100	Enterovirus B101	Enterovirus B106
Enterovirus B107	Enterovirus C109	Enterovirus C117				
**EV_ACD**						
Coxsackievirus A1	Coxsackievirus A11	Coxsackievirus A13	Coxsackievirus A17	Coxsackievirus A19	Coxsackievirus A20	Coxsackievirus A21
Coxsackievirus A22	Coxsackievirus A24	Enterovirus A121	Enterovirus A91	Enterovirus C96	Enterovirus C99	Enterovirus C102
Enterovirus D68	Enterovirus D70	Enterovirus D94	Enterovirus D111	Poliovirus 1	Poliovirus 2	Poliovirus 3
**RV_A**						
Rhinovirus A1	Rhinovirus A1B	Rhinovirus A2	Rhinovirus A7	Rhinovirus A8	Rhinovirus A9	Rhinovirus A10
Rhinovirus A11	Rhinovirus A13	Rhinovirus A15	Rhinovirus A16	Rhinovirus A18	Rhinovirus A19	Rhinovirus A20
Rhinovirus A21	Rhinovirus A22	Rhinovirus A25	Rhinovirus A28	Rhinovirus A29	Rhinovirus A30	Rhinovirus A31
Rhinovirus A32	Rhinovirus A33	Rhinovirus A34	Rhinovirus A36	Rhinovirus A38	Rhinovirus A39	Rhinovirus A40
Rhinovirus A41	Rhinovirus A43	Rhinovirus A44	Rhinovirus A46	Rhinovirus A47	Rhinovirus A49	Rhinovirus A50
Rhinovirus A53	Rhinovirus A54	Rhinovirus A56	Rhinovirus A57	Rhinovirus A58	Rhinovirus A59	Rhinovirus A60
Rhinovirus A61	Rhinovirus A62	Rhinovirus A63	Rhinovirus A64	Rhinovirus A66	Rhinovirus A67	Rhinovirus A68
Rhinovirus A73	Rhinovirus A74	Rhinovirus A75	Rhinovirus A76	Rhinovirus A77	Rhinovirus A80	Rhinovirus A81
Rhinovirus A82	Rhinovirus A85	Rhinovirus A88	Rhinovirus A89	Rhinovirus A90	Rhinovirus A94	Rhinovirus A95
Rhinovirus A96	Rhinovirus A98	Rhinovirus A100				

RV_B						
Rhinovirus B3	Rhinovirus B4	Rhinovirus B5	Rhinovirus B6	Rhinovirus B14	Rhinovirus B17	Rhinovirus B26
Rhinovirus B27	Rhinovirus B35	Rhinovirus B37	Rhinovirus B42	Rhinovirus B48	Rhinovirus B52	Rhinovirus B69
Rhinovirus B70	Rhinovirus B72	Rhinovirus B79	Rhinovirus B83	Rhinovirus B84	Rhinovirus B86	Rhinovirus B91
Rhinovirus B92	Rhinovirus B93	Rhinovirus B97	Rhinovirus B99			
**RV_C + EV_AC**						
Enterovirus C104	Enterovirus C118	Enterovirus A125	Rhinovirus C10	Rhinovirus C26	Rhinovirus C51	Rhinovirus C53
Rhinovirus C54	Rhinovirus C55					
**RV_AC**						
Rhinovirus A12	Rhinovirus A45	Rhinovirus A51	Rhinovirus A65	Rhinovirus A71	Rhinovirus A101	Rhinovirus A103
Rhinovirus C3	Rhinovirus C4	Rhinovirus C5	Rhinovirus C6	Rhinovirus C7	Rhinovirus C8	Rhinovirus C15
Rhinovirus C42	Rhinovirus C43					
